# C-reactive protein: quantitative marker of surgical trauma and post-surgical complications in dogs: a systematic review

**DOI:** 10.1186/s13028-015-0164-5

**Published:** 2015-10-20

**Authors:** Michelle B. Christensen, Thomas Eriksen, Mads Kjelgaard-Hansen

**Affiliations:** Department of Veterinary Clinical and Animal Sciences, Faculty of Health and Medical Sciences, University of Copenhagen, Dyrlaegevej 16, 1870 Frederiksberg C, Denmark; Novo Nordisk A/S, Novo Nordisk Park, 2760 Maaloev, Denmark

**Keywords:** C-reactive protein, Inflammation, Dog, Post-surgical infections, Surgical trauma

## Abstract

C-reactive protein (CRP) is a major acute phase protein showing increasing serum concentrations in dogs with systemic inflammation following e.g., surgery, trauma, infections, or neoplasia. CRP is a useful diagnostic marker of systemic inflammation in dogs and automated assays have been validated for reliable measurements for routine diagnostic purposes. In the present study available evidence for the use of CRP as a marker of surgery related systemic inflammation in dogs was reviewed and assessed. Two main themes were in focus: (1) canine CRP as a potential marker of postsurgical infectious complications and (2) canine CRP as a marker of the degree of surgical trauma. As outlined in the review several studies suggest that CRP is a useful marker for both purposes. However, the evidence level is limited and studies in the field are all affected by considerable risks of bias. Thus, further studies are needed in order to confirm the assumptions from previous studies and increase the level of evidence for CRP as a useful marker for detecting inflammation after surgery in dogs.

## Background

The acute phase response is a non-specific reaction to any tissue stimulation disturbing the homeostasis e.g., surgery, trauma, infection, or neoplasia [1–3] and plays an important role as part of the innate immune system [1, 3]. During the acute phase-response concentrations of acute phase proteins will change, reflecting the presence of circulating cytokines [1, 3, 4]. In dogs, C-reactive protein (CRP) is a major acute phase protein showing significant increases in concentration as a result of systemic inflammation [1–3, 5]. The function of CRP has been reviewed on several occasions, and includes suppression of microbial growth, clearance of damaged tissues, and regulation of the inflammatory response [1, 3, 6]. CRP is used as a diagnostic marker for detection of systemic inflammation in dogs [5, 7, 8] and quantitative measurements of canine CRP concentrations have been possible for more than 2 decades [9]. Several automated assays are validated for reliable diagnostic measurements of canine CRP and are used in clinical settings [10, 11]. CRP has become an integrated part of the routine blood panel analyzed at veterinary clinical pathology laboratories [5, 12] and inflammatory blood panels may be less optimal if measurements of CRP or other acute phase proteins are not included [13]. Compared to other markers of inflammation like body temperature and leukocyte counts, CRP has been suggested to be a more sensitive and reliable marker of systemic inflammation following surgery in dogs [9, 14]. Consequently, it has been suggested that routine measurements of CRP concentrations could improve the assessment of postoperative inflammation and clinical decision making during recovery after surgery in dogs [9].

This review evaluates CRP as a marker of surgery related systemic inflammation in dogs. Two clinically relevant intervention questions are discussed, in relation to the PICO components: population, intervention, comparison, and outcome, as recommended by O’Connor et al. [15]:

1. Does the serum CRP concentration deviate significantly in dogs with infectious complications post-surgery, when compared to dogs without such complications?
2. Does the serum CRP concentration reflect the degree of operative trauma following different categories of surgery?

## Search strategy and inclusion and exclusion criteria for references

The study was based on broad PubMed, Web of Science, Agris, and CAB Abstract database searches using the following key phrases: ‘(Canine OR dog) AND (CRP OR “C-reactive protein”) AND Surgery’. Only articles in English were included. Articles older than 25 years, articles not including dogs, not focused on surgery, or not including CRP measurements were subsequently excluded. Broad reviews of CRP not exclusively focusing on surgery and scientific abstracts were excluded. Bibliographies of relevant studies were searched and cross referenced to identify any additional studies relevant for inclusion. Data base searches were completed January the 28th 2015. The level of evidence of each study was objectively scored according to Harbour and Miller [16]. The risk of bias in each study was critically assessed based on the questions outlined in Table 1. Outcome assessment was based on quantitative CRP concentrations measured at sequential time-points after surgery.

**Table 1:** Questions answered in order to assess bias in each of the 29 studies

Selection bias	Were included dogs representative for the population of dogs?
Information bias	Were the criteria for classification of dogs in different groups defined and reported?
Performance bias	Were the researchers blinded from knowledge about the animals/groups when processing data?
Detection bias	Was the endpoint detectable? (e.g., was CRP measured at relevant time-points?)
Attrition bias	Were exclusion criteria defined and patients lost to follow up reported?
Reporting bias	Were reports free of suggestions of selective outcome reporting?
Other bias	Was the study apparently free of other problems that could result in high risk of bias?

The degree of operative trauma was arbitrarily categorized into three types of surgery. Thus, methods resulting in minor to moderate operative trauma (Category 1) were those requiring analgesia and rehabilitation support after surgery only to obtain full recovery, whereas methods resulting in major operative trauma were those also requiring advanced clinical care and post-surgical hospitalization (Category 2) [14]. Endoscopic procedures were categorized separately (Category 3), as minor inflammatory responses could be expected following such procedures compared to open surgery [14].

Complications included in the articles were critically assessed based on previously defined criteria for post-surgical complications in dogs [17].

## Review

**Fig. 1:**
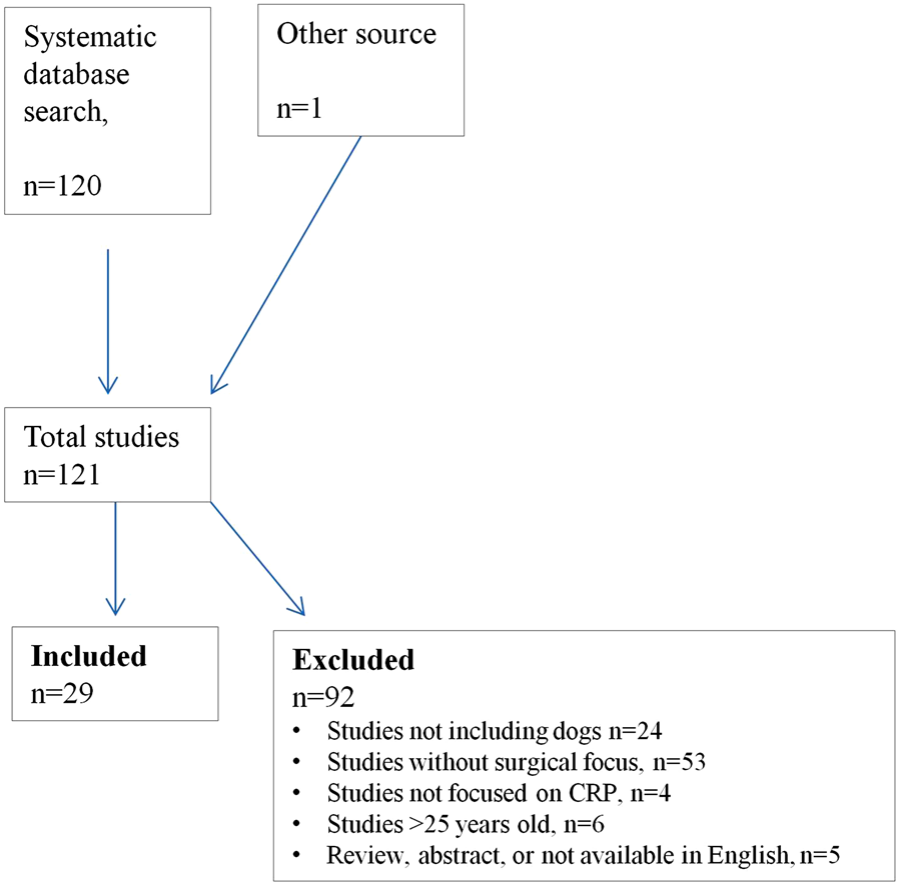
Flow-chart illustrating the inclusion and exclusion of studies in the systematic review also summarizing inclusion and exclusion criteria. The review was based on broad Pub Med, Web of Science, Agris, and CAB Abstract searches resulting in 120 hits, when duplicates were removed. Titles of studies and abstracts were analyzed for relevance. Bibliography of relevant studies were searched and cross referenced to identify any additional studies relevant for inclusion, thus identifying one additional study. Finally 29 articles were included

**Fig. 2:**
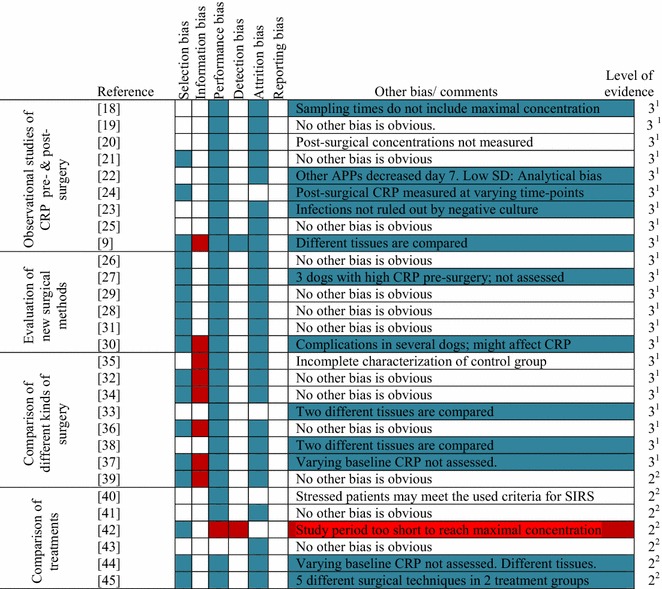
Risk of bias summary: Review authors’ judgments about each risk of bias item for each included study.* White boxes*: Low risk of bias (questions in Table 1 answered by ‘yes’). *Blue boxes*: Unclear risk of bias (questions in Table 1 could not be answered). *Red boxes*: High risk of bias (questions in Table 1 answered by ‘no’). Other bias/comments for which the authors assessed a consibarable risk of bias are marked with* grey* and further discussed in the text. Level of evidence was scored according to guidelines from Habour and Miller [16]: ^1^Observational studies, case-series. ^2^Qasi experimental studies, case-control, or cohort studies

Twenty-nine articles were included in this systematic review as illustrated in the flowchart (Fig. 1). The articles were divided in 4 groups according to their main objectives as illustrated in Table 2. Study sizes, surgical procedure, surgical category, and methods for measuring CRP concentrations used in the 29 publications are also summarized in Table 2. The studies included dogs undergoing ovariohysterectomy, ovarioectomy, or pyometra surgery (n = 14), different endoscopic procedures (n = 9), orchiectomy (n = 4), or other surgical procedures (balloon valvuloplasty, pericardiotomy, splenectomy, femoro-tibial joint surgery, tooth extractions, and excision of superficial tumours, n = 5), respectively. Male and female dogs with a wide range of bodyweight (4–64 kg) and age (3 months–14 years) were included in the studies, corresponding to the general population of dogs (Table 2). Most studies were observational or quasi experimental and were graded as evidence level 2 or 3 of 4, as defined by Habour and Miller [16] (Fig. 2). The risk of bias graph is shown in Fig. 2. Attrition, performance, and selection bias were the dominating types of bias, especially defined by insufficient information about inclusion and exclusion of dogs and blinding of researchers, but several studies were affected by other types of bias, which may affect the results even more (Fig. 2). No studies were assessed to be entirely free from bias and, consequently, all studies were assessed to have considerable risks of bias (Fig. 2).

**Table 2:** Overview of the 29 studies included in the review

Study focus	Reference	n	Sex, age (years), BW (kg)	Surgical procedure	Surgical category	Primary outcome	Pre-surgery inflammation	Method for CRP analysis
Observational studies of CRP measured post-surgery	[18]	27	F, 2–10, 5–30	Ovariohysterectomy	1	CRP	+	TIA^b^
	[19]	20	F, 2–4, 12–30	Ovariohysterectomy	1	CRP, WBC, Temp	−	ELISA^c^
	[20]	49	F, 0.75–12, 10–64	Ovariohysterectomy	1	CRP	−	TIA^d^
	[21]	5	M, 5–7, 14–21	Orchidectomy	1	CRP	−	TIA^b^
	[22]	6	F, 2–6, NS	Ovariohysterectomy	1	CRP	−	ELISA^c^
	[23]	20	F, 4–12, NS	Ovariohysterectomy	1	CRP	+	ELISA^c^
	[24]	16	F/M, 0.25–3, 4–39	Balloon valvuloplasty of pulmonic stenosis	1	CRP	−	ELISA^c^
	[25]	20	F, 3–12, NS	Ovariohysterectomy	1	CRP	±	ELISA^c^
	[9]	19	NS, NS, NS	5 different procedures	1 and 2	CRP	±	ELISA, RPLA^e^
Evaluation of new surgical methods	[26]	12	F/M, NS, 10–34	Double balloon endoscopy of intestine	3	Clinical outcome	−	NS
	[27]	12	NS, NS, 7–9	Endoscopic transumbilical thoracic lung biopsy	3	Clinical outcome	−	ELISA^f^
	[28]	14	NS, NS, 7–11	Transtracheal endoscopy of thorax	3	Clinical outcome	−	NS
	[29]	7	M, NS, 13.2^a^	Laparoscopic assisted colopexy and sterilization	3	Clinical outcome	−	IP^g^
	[30]	5	NS, NS, 20–28	Endoscopic ovariectomy	3	Clinical outcome	−	NS
	[31]	7	F, 2.5^a^, 9^a^	Laparoscopic-sutured gastropexy	3	CRP	−	ELISA^h^
Comparison of different kinds of surgery	[32]	28	NS, NS, 7–10	Transoral vs. conventional thoracoscopy	3	Perioperative stress	−	ELISA^f^
	[33]	32	F/M, 0.5–8, NS	Sterilization, 3 techniques	1 and 3	CRP	−	TIA^j^
	[34]	20	NS, NS, 6–11	Transoral vs. conventional thoracoscopy	2 and 3	Perioperative stress	−	ELISA^f^
	[35]	8	NS, 0.8–5, 15–25	Laparoscopic vs. laparotomic colopexy	1 and 3	Perioperative stress	−	ELISA^i^
	[36]	24	NS, adult, 25–30	Sternotomy and pericardiotomy vs. atriotomy	2	Perioperative stress	−	ELISA^b^
	[37]	30	F, NS, 11–38	Ovariectomy, 3 techniques	1 and 3	Perioperative stress	−	ELISA^h^
	[38]	43	F/M, >0.6, NS	Ovariohysterectomy vs. hemilaminectomy	1 and 2	CRP	±	TIA^j^
	[39]	15	F, NS, 17.4^a^	Splenectomy, 3 techniques	2 and 3	Perioperative stress	−	LAT^k^
Changes in CRP due to different treatments?	[40]	16	F, 3–14, 17.3^a^	Ovariohysterectomy	1	CRP	−	MIA^l^
	[41]	18	F, 1.5–4, 12–20	Ovariohysterectomy	1	CRP	−	ELISA^b^
	[42]	45	F, 2.7^a^, 11.9^a^	Ovariohysterectomy	1	CRP	−	ELISA^m^
	[43]	46	F/M, 2–3, 20.7^a^	Ovariohyster- and orchiectomy	1	Perioperative stress	−	ELISA^b^
	[44]	20	F/M, 9.5^a^, 16.3^a^	Ovariohyster- and orchiectomy	1	CRP	−	ELISA^b^
	[45]	12	F/M, 1.5–10, 12–45	Cruciate ligament rupture and patella luxation	2	Perioperative stress	+	ELISA^b^

C-reactive protein (CRP). *F* female dogs, *M* male dogs, *NS* not specified

^a^Mean. Surgical categories: *1* minor/moderate surgery, *2* major surgery, *3* endoscopic procedures

^b^Turbidimetric immunoassay, CRP OSR 6147 Olympus Life and Material Science Europe GMbH, Lismeehan, O’Callaghan’s Mills, Co., Clare, Ireland

^c^Enzyme-linked immunosorbent assay,Tridelta Development Limited, Kildare, Ireland

^d^Turbidimetric immunoassay, Randox Laboratories Ltd., Crumlin, UK

^e^RPLA: Reversed passive latex agglutination

^f^Enzyme linked immunosorbent assay, PharMingen, BD Biosciences, San Diago, USA

^g^Immunoprecipitation, Reagent Konelab ThermoElectron, Cergy Pontoise, France

^h^Enzyme linked immnosorbent assay, Life diagnostics, Inc, West Chester, USA

^i^Enzyme linked immunosorbent assay, unspecified

^j^Turbidimetric immunoassay, Bayer CRP TIA, Newbury, UK

^k^Latex agglutination turbidimetry, Huma Tex CRP, In Vitro Diagnostica, Itabira, Brazil

^l^Magnetic immunoassay, LifeAssays Canine CRP Test Kit, Sweden

^m^Enzyme linked immunosorbent assay, Alpco Diagnostics, Salem, NH, USA

16 of the 29 studies measured CRP concentrations as one of the primary outcome measures, whereas the remaining 13 studies included CRP concentrations as a secondary outcome measure, often as one of several parameters collectively assessing the perioperative stress response (Table 2). Consequently, the 16 studies measuring CRP concentrations as a primary outcome measure were considered the strongest for the particular focus of the present review.

### Changes in CRP as a predictor of prognosis

A single measurement of the pre-operative [20] or peak concentration of CRP is currently of limited value in the assessment of post-operative inflammation and thus the prognosis for the patient. As has been documented for canine sepsis [46], meningitis [47] and polyarthritis [48], changes in CRP concentration over time may be more useful for monitoring of, and prognostic prediction in, surgical patients [23, 38]. Thus, several studies have identified a role for routine post-operative CRP measurements in the detection of post-surgical complications [22, 25]. Increasing concentrations of CRP have been demonstrated as early as 6 h after surgery [29], and maximum concentrations have been observed after approximately 12–24 h [30, 38, 44]. Significantly increased concentrations of CRP have been demonstrated for several days after surgery [30, 31], followed by subsequent decline in CRP concentration as the homeostasis is gradually re-established during progression of normal postoperative healing [25]. The decreasing of the CRP approaching baseline concentrations have been demonstrated to happen at different time-points in different studies. While baseline concentrations were reached 1 week after surgery in one study [35] and 2 weeks after surgery in other studies [27–29], baseline concentrations were, however, still not reached after 17 days in yet another study [25]. Further studies are needed in order to explain these differences and to define the exact time-points for normalization of CRP concentrations post-surgery. In this process well-documented references for baseline CRP concentration would be helpful. Even though it has been demonstrated that baseline concentrations of CRP can be objectively established [49, 50], reference intervals or clinical decision limits were, however, not established in the studies, representing a possibility of bias. With well-established baseline CRP concentrations even small fluctuations in concentration could help detect minor inflammation and aid in clinical decision-making after surgery.

In the study of Serin and Ulutas [22] baseline concentrations were not reached 7 days after surgery, but might have been demonstrable later, if the study period had been extended. However, this study may be affected by analytical bias (Fig. 2) and results should therefore be assessed with caution. Haptoglobin and ceruloplasmin are other acute phase proteins measured in the study and the concentrations of these proteins are approximating baseline concentrations before CRP in this study [22], which is contradicting the normal understanding of the relative kinetics of these proteins [1], suggesting analytical bias (Fig. 2). Further, the standard deviations of CRP concentrations are lower in this study [22] compared to other studies using the same assay for CRP measurements. This could probably also be explained by analytical bias.

Uncontested of these risks of bias, persistently high or increasing concentrations of CRP have been suggested to indicate an ongoing inflammatory process, e.g., as a result of infection of the surgical wound [23]. To our knowledge, only one study has, however, investigated this in detail [23]. In this study, significant higher concentrations of CRP were found 3, 4, 7, 10, and 17 days post-surgery in dogs with infectious complications after surgery compared to dogs without such complications. In dogs with infectious complications, increasing concentrations of CRP were observed until the antimicrobial spectrum of the antibiotic treatment was widened, while decreasing concentrations of CRP were observed at similar time-points with discontinued antibiotic treatment in dogs with no signs of post-surgical infection [23]. Several definitions of surgical complications can be used in dogs [17]. In the study by Dabrowski et al. [23] infectious complications were verified by positive cultures [23], which might be useful as inclusion criteria for dogs with such complications. However, infectious complications were apparently not ruled out by negative cultures from dogs with ‘normal postoperative periods’ and this might be a source of bias in the study (Fig. 2). Consequently, further studies are needed in order to further explore the usefulness of sequential measurements of CRP as predictor of infectious complications after surgery in dogs.

### Changes in CRP as a predictor of operative trauma

Minimizing tissue trauma and subsequent stress response is a goal of surgery [42] and CRP is often included as an objective marker in studies investigating this response in dogs [37, 42, 43, 45]. CRP has on several occasions been used in studies investigating new surgical procedures for the veterinary clinic [26, 29, 31, 35], in studies using dogs as model for human disease [27, 28, 30], and in studies comparing different surgical procedures [32, 34]. In the last group several studies have compared surgical trauma by comparing different surgical procedures in different tissues [9, 33]. This approach may, however, represent a risk of bias (Fig. 2), as stimulation of different tissues might induce changes in the CRP concentration differently.

In studies investigating laparoscopic splenectomy, colopexy, and ovariohysterectomy with open surgery, significantly lower concentrations of CRP have been demonstrated in laparoscopic surgery compared to open surgery in comparable tissues [33, 35, 39]. However, CRP was measured as a secondary outcome measure in most of these studies (Table 2) and all studies were affected by considerable risks of bias (Fig. 2). The results of the studies should, consequently be interpreted with caution with regard to CRP.

**Fig. 3:**
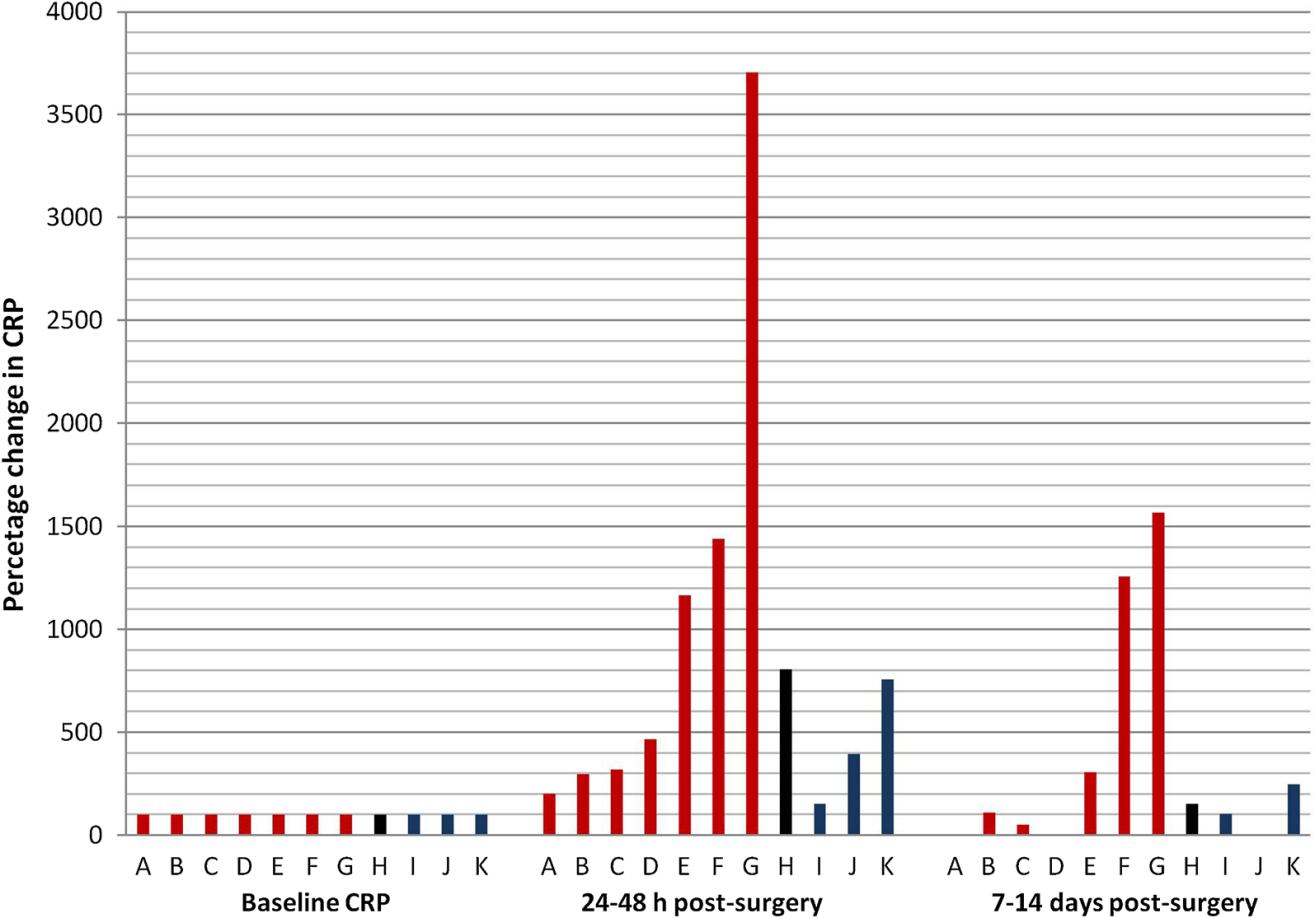
Relative post-surgical changes in C-reactive protein (CRP) at 24–48 h and 7–14 days following different kinds of surgery previoulsly published as ‘case-series’. Relative changes were calculated from mean or median concentrations of CRP before and after surgery. Baseline concentrations were plotted as 100 and post-surgical concentrations were calculated as percentage of baseline concentrations. Different types of surgeries were categrorized as defined in the text: Category 1 (*red columns*, reqiring minor analgetic and rehabilitating support after surgery): Tooth extraction (‘case series’ *A*, [9], n = 4), open colopexy (‘case series’ *B*, [35], n = 8), excision of superficial tumour (‘case series’ *C*, [9], n = 15), baloon valvuloplasty (‘case series’ *D*, [24], n = 15), and ovariohysterectomy (‘case series’ *E*–*G*, [9, 19, 22], n = 3–20). Category 2 (*black columns*, reqiring specialist clinical care and post-surgical hospitalization): Orthopedic surgery (‘case series’ *H*, [9], n = 4). Category 3 (*blue columns*, endoscopic procedures): Laparoscopic colopexy (‘case series’ *I*, [35], n = 8), laparoscopic gastropexy (‘case series’ *J*, [31], n = 7), and laparoscopic colopexy and vasectony (‘case series’ *K*, [29], n = 7)

**Fig. 4:**
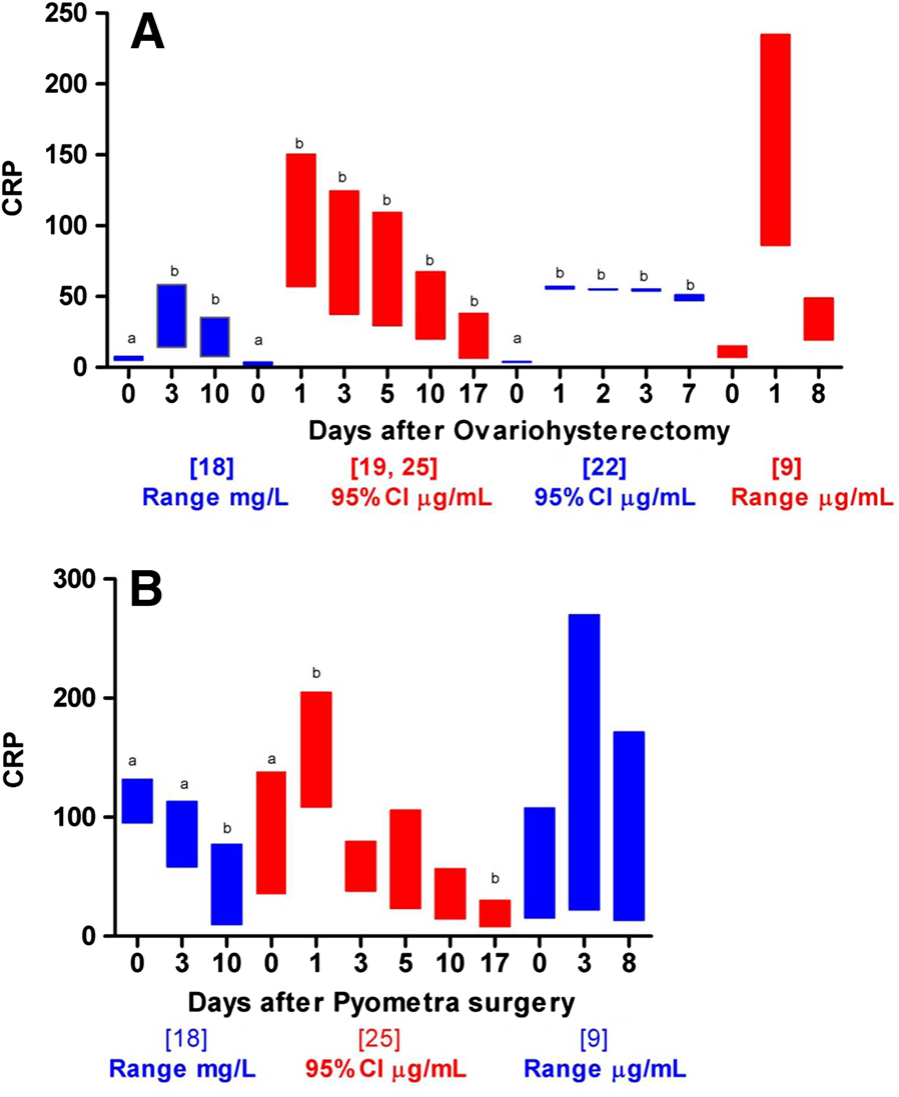
C-reactive protein (CRP) concentrations in dogs before and at several time-points after **A** elective ovariohysterectomy and **B** surgical treatment of pyometra [9, 18, 19, 22, 25]. Different superscription letters (*a*, *b*) represents significant differences between concentrations at different time-points (P < 0.05). When possible 95 % confidence intervals (CI) were calculated from means and standard deviations stated in the studies. When information for 95 % CI calculations were not available, concentration ranges were plotted instead

When changes in CRP concentrations were compared with operative trauma across studies and surgical procedures, considerable variations were observed, which could not be explained by different levels of surgical trauma (Fig. 3). Thus, measured concentrations of CRP did not reflect the category of surgical trauma (Fig. 3). This was also the case when dogs were compared following ovariohysterectomy representing a homogenous surgery group, as varying concentrations of CRP were observed (Figs. 3, 4). Several possible explanations for this variation should be considered.

One important factor could be the wide variety of quantitative methods used for measuring CRP concentrations across the 29 studies included in the review (Table 2). Even when using the same method for measuring CRP concentrations, varying concentrations could be expected across different laboratories and assay batches, particularly if the method was based on polyclonal human antibodies and calibrated with human CRP [12]. The different methods and laboratories used across the 29 studies could, therefore, explain some of the observed differences in CRP concentrations at specific time-points following similar surgical procedures (Figs. 3, 4). The recent availability of a novel canine CRP assay based on anti-canine CRP antibodies should limit this effect in future studies ensuring stable cross-reactivity with canine CRP across different assay batches [10]. However, significant differences across CRP analyses should be expected when older studies are reviewed.

Pre-surgical inflammation, resulting in increased CRP concentrations before surgery (Table 2), could be another important explanation of varying changes in CRP concentrations, not reflecting expected degrees of surgical trauma (Figs. 3, 4). Elevated concentrations of CRP before surgery, e.g., as a result of trauma, as observed in some orthopedic patients, or infections as observed in patients suffering from pyometra, may only result in limited, additional increases post-surgery. Elevated baseline concentrations of CRP were, thus, observed in dental, orthopedic, elective ovariohysterectomy, and cancer patients included in the study of Yamamoto et al. [9] and in 3 dogs before endoscopic transumbilical thoracic surgical lung biopsy in the study of Wen et al. [27], but as no other information regarding the level of preoperative systemic inflammation was given in these publications, the exact cause of these elevations are not known. This kind of bias was avoided in most studies investigating CRP following ovariohysterectomy or pyometra. Thus, predefined criteria were used to ensure that included dogs were clinically healthy before elective ovariohysterectomy [18, 19, 22, 25], while in studies of pyometra, the diagnosis was often confirmed by postsurgical histopathology [18, 20]. However, despite such precautions and standardized surgical procedures, significantly varying CRP concentrations were measured across these studies (Figs. 3, 4).

Another factor, which could explain that changes in CRP concentration was not reflecting the degree of operative trauma (Fig. 3), could be the level of surgeon experience. It has been speculated that higher concentrations of CRP may be expected in dogs following procedures performed by inexperienced surgeons, probably as a result of increased surgery time and tissue trauma in dogs operated by inexperienced surgeons compared to experienced surgeons [42]. This factor should, therefore, be considered, when investigating the use of CRP as a marker after surgery. Information about the surgeons’ experience was, however, only available in 5 studies [38, 41–43, 45], and the influence of the surgeons’ experience on the observed differences in CRP concentrations in the present review is therefore not known. However, further studies are needed in order to investigate the importance and influence of surgical experience on post-surgical CRP concentrations. Because of the prolonged surgery time when surgery was performed by inexperienced surgeons, follow-up CRP was measured at a later time-point relatively to start of surgery [42]. Consequently, higher concentrations could be expected 6 h after surgery performed by inexperienced surgeons, because of an extended time-period from initiation of surgery to measurement of CRP. In order to give more useful information about the degree of surgical trauma in the two groups, maximum concentrations should be compared. As maximum CRP concentrations cannot be expected 6 h after surgery the study period should be extended in order to reach this goal. The limited study period in this study, thus, represents a possible source of bias (Fig. 2).

On the other hand, differences in anesthesia do only seem to affect CRP concentrations to a limited degree. In one study CRP was, thus, not induced in 6 dogs exposed to anesthesia alone [36], and no differences in CRP concentrations have been demonstrated following varying use of epidural analgetics [45], non-steroid anti-inflammatory drugs [41], or dog-appeasing pheromone [43]. Even though anesthetic protocols varied significantly across the 29 studies included in the review, these differences should, therefore, not affect measured concentrations of CRP. One study has, however, shown significant lower concentrations of CRP as a result of treatment with low doses of ketamine during and after surgery [40], as also demonstrated in humans [51]. Ketamine was included in the anesthetic protocols of 6 studies included in the review [22, 27, 28, 32, 34, 41], and CRP concentrations in these studies could potentially be affected when compared to the remaining studies. However, other studies in human medicine have shown conflicting results [52, 53] and the conclusions for dogs were all based on a single study affected by considerable risks of bias (Fig. 2). Therefore, further studies are needed to confirm the results and increase the level of evidence.

## Conclusions

CRP might be useful as a marker of systemic inflammation after surgery in dogs, but currently the evidence level is limited and affected by considerable risks of bias. Thus, it is not possible to answer the PICO questions asked above with the current level of evidence:

1. The serum CRP concentration may deviate significantly in dogs with infectious complications post-surgery, when compared to dogs without such complications, but more evidence is needed to confirm these assumptions and to establish criteria for the differentiation of infections from normal post-surgical changes.
2. The serum CRP concentration may reflect the degree of operative trauma following different categories of surgery, but comparable studies based on comparable study designs and comparable assays for CRP measurements are needed to increase the evidence in this field.

## References

[1] Ceron J, Eckersall P, Martinez-Subiela S (2005). Acute phase proteins in dogs and cats: current knowledge and future perspectives. Vet Clin Pathol.

[2] Eckersall PD, Bell R (2010). Acute phase proteins: biomarkers of infection and inflammation in veterinary medicine. Vet J..

[3] Cray C, Zaias J, Altman NH (2009). Acute phase response in animals: a review. Comp Med.

[4] Petersen H, Nielsen J, Heegaard P (2004). Application of acute phase protein measurements in veterinary clinical chemistry. Vet Res.

[5] Kjelgaard-Hansen M, Jacobsen S (2011). Assay validation and diagnostic applications of major acute-phase protein testing in companion animals. Clin Lab Med..

[6] Murata H, Shimada N, Yoshioka M (2004). Current research on acute phase proteins in veterinary diagnosis: an overview. Vet J..

[7] Eckersall PD, Schmidt EM (2014). The final hurdles for acute phase protein analysis in small animal practice. J Small Anim Pract.

[8] Nakamura M, Takahashi M, Ohno K, Koshino A, Nakashima K, Setoguchi A, Fujlno Y, Tsujimoto H (2008). C-reactive protein concentration in dogs with various diseases. J Vet Med Sci.

[9] Yamamoto S, Shida T, Miyaji S, Santsuka H, Fujise H, Mukawa K (1993). Changes in serum C-reactive protein-levels in dogs with various disorders and surgical traumas. Vet Res Commun.

[10] Hillström A, Hagman R, Tvedten H, Kjelgaard-Hansen M (2014). Validation of a commercially available automated canine-specific immunoturbidimetric method for measuring canine C-reactive protein. Vet Clin Path..

[11] Klenner S, Bauer N, Moritz A (2010). Evaluation of three automated human immunoturbidimetric assays for the detection of C-reactive protein in dogs. J Vet Diagn Invest.

[12] Kjelgaard-Hansen M (2010). Comments on measurement of C-reactive protein in dogs. Vet Clin Path..

[13] Ceron JJ, Martinez-Subiela S, Ohno K, Caldin M (2008). A seven-point plan for acute phase protein interpretation in companion animals. Vet J..

[14] Watt D, Horgan P, McMillan D (2015). Routine clinical markers of the magnitude of the systemic inflammatory response after elective operation: a systematic review. Surgery.

[15] O’Connor AM, Anderson KM, Goodell CK, Sargeant JM (2014). Conducting systematic reviews of intervention questions I: writing the review protocol formulating the question and searching the literature. Zoonoses Publ Health..

[16] Habour R, Miller J (2001). A new system for grading recommendations in evidence based guidelines. BMJ.

[17] Eugster S, Schawalder P, Gaschen F, Boerlin P (2004). A prospective study of postoperative surgical site infections in dogs and cats. Vet Surg.

[18] Dabrowski R, Szczubiał M, Kostro K, Wawron W, Ceron JJ, Tvarijonaviciute A (2015). Serum insulin-like growth factor-1 and C-reactive protein concentrations before and after ovariohysterectomy in bitches with pyometra. Theriogenology.

[19] Dabrowski R, Wawron W (2014). Acute-phase response in monitoring postoperative recovery in bitches after overiohysterectomy. Ann Anim Sci..

[20] Jitpean S, Holst B, Höglund OV, Pettersson A, Olsson U, Strage FS, Hagman R (2014). Serum insulin-like growth factor-I, iron, C-reactive protein, and serum amyloid A for prediction of outcome in dogs with pyometra. Theriogenology.

[21] Tvarijonaviciute A, Martinez-Subiela S, Carrillo-Sanchez JD, Tecles F, Ceron JJ (2011). Effects of orchidectomy in selective biochemical analytes in Beagle dogs. Reprod Dom Anim..

[22] Serin G, Ulutas P (2010). Measurement of serum acute phase proteins to monitor postoperative recovery in anoestrous bitches after overiohysterectomy. Vet Rec..

[23] Dąbrowski R, Kostro K, Lisiecka U, Szczubiał M, Krakowski L (2009). Usefulness of C-reactive protein, serum amyloid A component, and haptoglobin determinations in bitches with pyometra for monitoring early post-ovariohysterectomy complications. Theriogenology.

[24] Saunders AB, Smith BE, Fosgate GT, Suchodolski JS, Steiner JM (2009). Cardiac troponin I and C-reactive protein concentrations in dogs with severe pulmonic stenosis before and after balloon valvuloplasty. J Vet Card..

[25] Dabrowski R, Wawron W, Kostro K (2007). Changes in CRP, SAA and haptoglobin produced in response to ovariohysterectomy in healthy bitches and those with pyometra. Theriogenology.

[26] Sarria R, Albors OL, Soria F, Ayala I, Cuadrado EP, Esteban P, Latorre R (2013). Characterization of oral double balloon endoscopy in the dog. Vet J..

[27] Wen C, Chu Y, Yeh C, Liu C, Yuan H, Ko P (2013). Feasibility and safety of endoscopic transumbilical thoracic surgical lung biopsy: a survival study in a canine model. J Surg Res.

[28] Liu Y, Chu Y, Wu Y, Yeh C, Chang H, Ko P, Liu H (2011). Natural orifice transluminal endoscopic surgery: a transtracheal approach for the thoracic cavity in a live canine model. J Thoracic Cardiovasc. Surg..

[29] Mathon D, Palierne S, Meynaud-Collard P, Layssol-Lamour C, Dulaurent-Ferrieres A, Colson A, Lacroix M, Bousquet-Melou A, Delverdier M, Autefage A (2011). Laparoscopic-assisted colopexy and sterilization in male dogs: short-term results and physiologic consequences. Vet Surg.

[30] Freeman LJ, Rahmani EY, Sherman S, Chiorean MV, Selzer DJ, Constable PD, Snyder PW (2009). Oophorectomy by natural orifice transluminal endoscopic surgery: feasibility study in dogs. Gastroint Endosc..

[31] Mathon DH, Dossin O, Palierne S, Cremoux M, Rodriguez H, Meynaud-Collard P (2009). A laparoscopic-sutured gastropexy technique in dogs: mechanical and functional evaluation. Vet Surg.

[32] Chu Y, Liu C, Wu Y, Hsieh M, Chen T, Chao Y (2013). Comparison of hemodynamic and inflammatory changes between transoral and transthoracic thorascopic surgery. PLoS One.

[33] Kjelgaard-Hansen M, Strom H, Mikkelsen LF, Eriksen T, Jensen AL, Luntang-Jensen M (2013). Canine serum C-reactive protein as a quantitative marker of the inflammatory stimulus of aseptic elective soft tissue surgery. Vet Clin Path..

[34] Liu C, Chu Y, Wu Y, Yuan H, Ko P, Liu Y, Liu H (2013). Transoral endoscopic surgery versus conventional thoracoscopic surgery for thoracic intervention: safety and efficacy in a canine survival model. Surg Endosc.

[35] Zhang S, Wang H, Zhang J, Zhang N, Pan L (2013). Laparoscopic colopexy in dogs. J Vet Med Sci.

[36] Schuessler R, Ishii Y, Khagi Y, Diabagate K, Boineau J, Damiano R (2012). The effect of inflammation on heart rate and rhythm in a canine model of cardiac surgery. Heart Rhythm..

[37] Freeman LJ, Rahmiani EY, Al-Haddad M, Sherman S, Chiorean MV, Selzer DJ, Snyder PW, Constable PD (2010). Comparison of pain and postoperative stress in dogs undergoing natural orifice transluminal endoscopic surgery, laparoscopic, and open oophorectomy. Gastroint Endosc..

[38] Nevill B, Leisewitz A, Goddard A, Thompson P (2010). An evaluation of changes over time in serum creatinine kinase activity and C-reactive protein concentration in dogs undergoing hemilaminectomy or ovariohysterectomy. J South Afr Vet Assoc..

[39] Stedile R, Beck CAC, Schiochet F, Ferreira MP, Oliveira S, Martens FB (2009). Laparoscopic versus open splenectomy in dogs. Pesq Vet Bras..

[40] Liao P, Chang S, Chen K, Wang H (2014). Decreased postoperative C-reactive protein production in dogs with pyometra through the use of low-dose ketamine. J Vet Emerg Crit Care..

[41] Kum C, Voyvoda H, Sekkin S, Karademir U, Tarimcilar T (2013). Effects of carprofen and meloxicam on C-reactive protein, ceruloplasmin, and fibrinogen concentrations in dogs undergoing ovariohysterectomy. Am J Vet Res.

[42] Michelsen J, Heller J, Wills F, Noble GK (2012). Effect of surgeon experience on postoperative plasma cortisol and c-reactive protein concentrations after overiohysterectomy in the dog: a randomised trial. Austr. Vet. J..

[43] Siracusa C, Manteca X, Cuenca R, Alcalá M, Alba A, Lavín S, Pastor J (2010). Effect of a synthetic appeasing pheromone on behavioral, neuroendocrine, immune, and acute-phase perioperative stress responses in dogs. J Am Vet Med Assoc.

[44] Saunders AB, Hanzlicek AS, Martinez EA, Stickney MJ, Steiner JM, Suchodolski JS, Fosgate GT (2009). Assessment of cardiac troponin I and C-reactive protein concentrations associated with anesthetic protocols using sevoflurane or a combination of fentanyl, midazolam and sevoflurane in dogs. Vet Anaesth Analg..

[45] Sibanda S, Hughes J, Pawson P, Kelly G, Bellenger C (2006). The effect of preoperative extradural bupivacaine and morphine on the stress response in dogs undergoing femoro-tibial joint surgery. Vet Anaesth Analg.

[46] Gebhardt C, Hirschberger J, Rau S, Arndt G, Krainer K, Schweigert FJ (2009). Use of C-reactive protein to predict outcome in dogs with systemic inflammatory response syndrome or sepsis. J Vet Emerg Crit Care..

[47] Lowrie M, Penderis J, McLaughlin M, Eckersall PD, Anderson TJ (2009). Steroid responsive meningitis-arteritis: a prospective study of potential disease markers, prednisolone treatment, and long-term outcome in 20 dogs (2006–2008). J Vet Int Med..

[48] Kjelgaard-Hansen M, Jensen AL, Houser GA, Jessen LR, Kristensen AT (2006). Use of serum C-reactive protein as an early marker of inflammatory activity in canine type II immune-mediated polyarthritis: case report. Acta Vet Scand.

[49] Kjelgaard-Hansen M, Mikkelsen LF, Kristensen AT, Jensen AL (2003). Study on biological variability of five acute-phase reactants in dogs. Comp Clin Path..

[50] Christensen MB, Langhorn R, Goddard A, Andreasen E, Moldal E, Tvarijonaviciute A (2014). Comparison of serum amyloid A and C-reactive protein as markers of systemic inflammation in dogs. Can Vet J.

[51] Bhutta A, Schmitz M, Swearingen C, James L, Wardbegnoche W, Lindquist D (2012). Ketamine as a neuroprotective and anti-inflammatory agent in children undergoing surgery on cardiopulmonary bypass: a pilot randomized, double-blind, placebo-controlled trial. Pediatr Crit Care Med..

[52] Cho J, Shim J, Choi Y, Kim D, Hong S, Kwak Y (2009). Effect of low-dose ketamine on inflammatory response in off-pump coronary artery bypass graft surgery. Br J Anaesth.

[53] D’Alonzo R, Bennett-Guerrero E, Podgoreanu M, D’Amico T, Harpole D, Shaz A (2011). A randomized, double blind, placebo controlled clinical trial of the preoperative use of ketamine for reducing inflammation and pain after thoracic surgery. J Anesth..

